# Impact of a multimodal prehabilitation program on postoperative recovery in patients undergoing total laparoscopic hysterectomy and its clinical effects on reducing the risk of venous thromboembolism occurrence

**DOI:** 10.3389/fonc.2026.1842401

**Published:** 2026-09-16

**Authors:** Liuhua Qu, Ling Yang, Lu Li

**Affiliations:** Department of Gynaecology, Ningyuan County People’s Hospital, Yongzhou, Hunan, China

**Keywords:** enhanced recovery after surgery, hysterectomy, laparoscopy, postoperative complications, venous thromboembolism

## Abstract

**Background:**

Postoperative recovery and venous thromboembolism (VTE) are major concerns following total laparoscopic hysterectomy (TLH). This study aimed to evaluate the impact of a multimodal prehabilitation program on postoperative recovery and its clinical effects on reducing VTE risk in patients undergoing TLH.

**Methods:**

This retrospective study involved 274 patients who underwent TLH for benign uterine diseases. Patients were divided into a conventional group (n = 145) and a prehabilitation group (n = 129). Postoperative recovery indicators, Caprini risk scores, lower extremity deep vein thrombosis (DVT) incidence, serum markers (coagulation function and inflammatory parameters), and complications were compared between groups.

**Results:**

Compared with the conventional group, patients in the prehabilitation group demonstrated significantly shorter times to first anal exhaust (22.63 ± 4.38 vs. 24.47 ± 5.44 h, P = 0.002), first bowel movement (61.08 ± 6.23 vs. 63.26 ± 7.21 h, P = 0.008), first ambulation (18.45 ± 3.74 vs. 22.34 ± 4.09 h, P < 0.001), and postoperative hospital stay (4.39 ± 0.71 vs. 6.95 ± 1.03 days, P < 0.001). Postoperative pain scores at 6, 24, and 48 h were also significantly lower in the prehabilitation group (all P < 0.05). Additionally, the prehabilitation group showed improved postoperative Caprini risk profiles (P < 0.001) and significantly lower incidence of lower-extremity DVT (2.33% vs. 14.48%, P < 0.001). Coagulation function and inflammatory markers were also significantly improved in the prehabilitation group. Multivariate analysis further identified the prehabilitation program as an independent protective factor against postoperative DVT (OR = 0.223, 95%CI: 0.099–0.502, P < 0.001).

**Conclusion:**

Multimodal prehabilitation significantly enhances postoperative recovery and reduces the risk of venous thromboembolism in patients undergoing TLH.

## 1 Introduction

Total laparoscopic hysterectomy (TLH) has become a standard surgical procedure for benign uterine conditions, including fibroids, adenomyosis, and endometriosis, due to its remarkable advantages over traditional open surgery, such as reduced intraoperative blood loss, shorter hospital stays, and faster return to daily activities (1, 2). However, despite the minimally invasive benefits of TLH, patients remain susceptible to a spectrum of postoperative challenges that can delay recovery and increase healthcare burden (3, 4).

Among these challenges, VTE, which encompasses deep vein thrombosis (DVT) and pulmonary embolism, is a particularly serious and potentially life-threatening complication (5). The pathogenesis of postoperative VTE is multifactorial and mainly explained by Virchow’s triad: venous stasis, endothelial injury, and hypercoagulability. Factors such as prolonged surgical positioning, increased intra-abdominal pressure due to pneumoperitoneum, and the systemic inflammatory response to surgical trauma collectively increase the risk of thrombotic events in patients undergoing gynecologic surgery (6, 7). The reported incidence of DVT after major gynecologic surgery without prophylaxis ranges from 15% to 40%, highlighting the critical need for effective preventive strategies (5). Although current perioperative guidelines recommend mechanical and pharmacological thromboprophylaxis, the optimal timing and comprehensive integration of these measures remain areas of ongoing investigation (8).

Beyond the specific risk of VTE, postoperative recovery is complex and multifaceted, encompassing the restoration of gastrointestinal function, effective pain management, early ambulation, and prevention of complications (9). Delayed recovery not only prolongs hospital stays and increases healthcare costs but also negatively impacts patients’ quality of life and psychological well-being (10). In this context, the Enhanced Recovery After Surgery (ERAS) paradigm has gained widespread recognition as a multimodal, evidence-based perioperative care strategy designed to accelerate recovery and reduce morbidity (11). However, traditional ERAS protocols have predominantly focused on intraoperative and postoperative interventions, with comparatively less attention directed toward preoperative optimization of patients’ physiological reserve (12).

This recognition has stimulated growing interest in prehabilitation, a proactive, preoperative intervention strategy designed to enhance functional capacity and prepare patients to successfully withstand surgical stress. Multimodal prehabilitation typically integrates exercise training, nutritional optimization, and psychological support, addressing the interconnected domains of physical, metabolic, and emotional health (13). Emerging evidence indicates that such preoperative conditioning may improve postoperative outcomes across various surgical disciplines (14). In gynecologic surgery, preliminary studies have demonstrated benefits in reducing postoperative complications and shortening hospital stay. However, a specific gap exists: the impact of a structured multimodal prehabilitation program that incorporates preoperative thromboprophylaxis on VTE incidence and overall recovery parameters in patients undergoing TLH for benign diseases has not been systematically evaluated (15). The present patient population (benign uterine diseases, low-to-moderate anesthetic risk) allows assessment of prehabilitation effects without the confounding influence of malignancy-related hypercoagulability or extensive comorbidities.

Therefore, this study aimed to examine the clinical effects of a multimodal prehabilitation program on postoperative recovery and its role in reducing the risk of VTE in patients undergoing TLH for benign diseases.

## 2 Materials and methods

### 2.1 Research cases

A retrospective analysis was conducted on 274 patients who underwent TLH at our hospital between January 2023 and December 2025. The inclusion criteria were as follows: (a) age ≥18 years; (b) preoperative diagnosis of benign uterine diseases (uterine fibroids, adenomyosis, and endometriosis), confirmed by postoperative pathology; (c) American Society of Anesthesiologists (ASA) (16) classification I–II; and (d) complete medical records. The exclusion criteria included the following: (a) cervical or endometrial malignancy; (b) history of previous abdominal surgery; (c) history of prior VTE; (d) severe heart, lung, liver, or kidney dysfunction; (e) coagulation disorders; (f) cognitive impairment or psychiatric disorders; and (g) use of anticoagulant or hormone medications within four weeks before surgery.

Patients were divided into two groups based on the nursing program they received: a conventional group (n = 145) and a prehabilitation group (n = 129). The conventional group consisted of consecutive patients who underwent TLH between January 2023 and June 2024, before the multimodal prehabilitation program was implemented. The prehabilitation group comprised consecutive patients who underwent TLH between July 2024 and December 2025, after the program had been integrated into routine clinical practice.

The protocol for this retrospective clinical study was reviewed and approved by the Medical Ethics Committee of Ningyuan County People’s Hospital (Approval Number: 2026-2). Given the retrospective design of this study, which does not interfere with the original treatment plans of patients and does not impose any additional medical risks or financial burdens during data extraction, the requirement for informed consent was waived after approval by the ethics committee.

### 2.2 Nursing programs

The conventional group received postoperative standard care: (a) mechanical prophylaxis: wearing compression stockings and using intermittent pneumatic compression therapy within 6 h after surgery; (b) pharmacological prophylaxis: guided by the Caprini risk assessment model, high-risk patients received low-molecular-weight heparin (LMWH) within 24 h after surgery for 7–14 days postoperatively, while very high-risk patients extended the treatment to four weeks; (c) early mobilization: assisting patients to ambulate within 24 h after surgery and gradually increasing the amount of physical activity.

In addition to standard care, the prehabilitation group received additional preoperative multimodal prehabilitation. This program comprised four components: (a) thrombosis risk assessment—high-risk and very-high-risk patients received LMWH prophylaxis 24 hours before surgery, based on the Caprini risk model; (b) exercise training—patients performed ankle pump exercises (10 repetitions per hour while awake), respiratory training using incentive spirometry (5–10 breaths every 2 hours), and a progressive walking program (at least 30 minutes daily) to enhance circulation; (c) nutritional support—we corrected anemia (target hemoglobin ≥100 g/L) and optimized nutritional status with vitamin D (800 IU/day) and protein supplementation (1.2–1.5 g/kg ideal body weight/day); (d) psychological intervention—we delivered health education via videos and manuals in two 20-minute sessions (one week before surgery and on the day before surgery) to reduce anxiety and improve compliance. All exercise training (ankle pumps, respiratory training, walking) and psychological interventions (education videos/manuals) were administered exclusively during the preoperative period. The prehabilitation program was initiated 7 days before the scheduled surgery date. All 129 patients in the prehabilitation group received the full protocol, as documented in standardized preoperative order sets and nursing records. The completeness of recorded interventions was independently verified by two data abstractors (inter-rater agreement of 98.6%).

All components—exercise training, nutritional support, psychological intervention, and preoperative LMWH prophylaxis for high/very high-risk patients—were delivered as an integrated, multimodal bundle. In the prehabilitation group, high-risk and very-high-risk patients received LMWH prophylaxis starting 24 hours before surgery as an integral component of the multimodal program. In contrast, patients in the conventional group initiated LMWH postoperatively (within 24 h after surgery), in accordance with standard perioperative guidelines. This difference in LMWH timing is perfectly collinear with group allocation and therefore inseparable from the composite intervention effect.

### 2.3 Outcome assessments

#### 2.3.1 Postoperative recovery

Data collected for both groups included time to first postoperative anal exhaust, first bowel movement, first postoperative ambulation, and postoperative hospital stay. Additionally, pain levels were assessed using the visual analogue scale (VAS) at the following time points: preoperatively, and at 6, 24, and 48 h postoperatively. VAS scores ranged from 1 (no pain) to 10 (worst imaginable pain), with an intraclass correlation coefficient of 0.99 (17).

#### 2.3.2 VTE

The Caprini risk assessment scale [3] was performed preoperatively and repeated 24 h postoperatively to evaluate dynamic changes in thrombotic risk attributable to surgical factors and the intervention. This scale includes 40 risk factors, each assigned different scores (1, 2, 3, or 5 points) based on their association with VTE. The total score for each patient was calculated. Patients were categorized into four risk levels: low risk (0–1 point), moderate risk (2 points), high risk (3–4 points), and very high risk (≥5 points), with a kappa value of 0.93 (18). Additionally, the incidence of lower-extremity DVT was assessed in both groups. All patients routinely underwent lower-extremity color Doppler ultrasound (Acuson S2000, Siemens Healthcare, Germany) between postoperative days 3 and 7. If patients exhibited suspicious DVT symptoms, such as leg swelling or pain during hospitalization or within 30 days after discharge, then additional ultrasound examinations were performed. DVT was diagnosed based on the presence of any of the following conditions were: non-tender venous lumen, absence of respiratory variation in Doppler pulse spectra, anechoic or hypoechoic appearance within the venous lumen, and absence of blood flow signals in venous segments (19). All 274 patients completed the 30-day postoperative follow-up (clinic visits or structured telephone interviews). No patients were lost to follow-up, and data for the reported variables were all available.

#### 2.3.3 Serum indicators

We measured activated partial thromboplastin time (APTT), prothrombin time (PT), fibrinogen (FIB), D-dimer (D-D), C-reactive protein (CRP), and interleukin-6 (IL-6) preoperatively and 24 hours postoperatively. We used an automated coagulation analyzer (CA-2000i, Sysmex, Japan) and a fully automated biochemical analyzer (7600, Hitachi High-Tech, Japan) for these measurements.

#### 2.3.4 Complications

The incidence of postoperative complications, including wound infection, urinary tract infection, subcutaneous emphysema, and shoulder-back pain, was recorded.

### 2.4 Statistical analyses

Statistical analyses were performed using SPSS 29.0 statistical software (SPSS Inc., Chicago, IL, USA). A two-tailed significance level of α = 0.05 was applied, and P < 0.05 was considered statistically significant. Initially, the Shapiro–Wilk test was used to assess the normality of continuous variables, all of which were confirmed to follow a normal distribution and are presented as mean ± standard deviation (M ± SD). Between-group comparisons of continuous variables were conducted using independent-samples t-tests. Categorical variables were expressed as frequencies and percentages [n (%)] and were compared between groups using the χ² test. Univariate and multivariate logistic regression analyses were performed with postoperative lower-extremity DVT occurrence as the dependent variable (no occurrence = 0, occurrence = 1) to identify independent risk factors for postoperative DVT in patients undergoing TLH. Variables with a P-value < 0.10 in the univariate analysis were considered candidates for entry into the multivariate logistic regression model. Multivariate modeling was performed using a forward stepwise selection procedure with an entry criterion of P < 0.05 and a removal criterion of P > 0.10. Collinearity among covariates was assessed using variance inflation factors (VIF), and all retained variables demonstrated VIF < 5, indicating no significant multicollinearity. The final model was adjusted for variables that met the selection criteria. Results are presented as odds ratios (OR) with 95% confidence intervals (CI).

## 3 Results

### 3.1 Baseline characteristics

Comparison of baseline characteristics between the conventional group (n = 145) and the prehabilitation group (n = 129) for patients undergoing TLH revealed no significant differences in age, BMI, smoking status, drinking habits, hypertension prevalence, diabetes incidence, ASA classification distribution, or disease type distribution (all P > 0.05). These findings indicate that the two groups were comparable at baseline (**Table 1**).

**Table 1:** Comparison of baseline characteristics between two groups.

Parameter	Conventional group (n=145)	Prehabilitation group (n=129)	t/χ^2^	P
Age (years)	49.95 ± 4.37	50.54 ± 5.08	1.027	0.305
BMI (kg/m^2^)	24.63 ± 3.21	24.98 ± 3.45	0.876	0.382
Smoking [n(%)]	9 (6.21%)	10 (7.75%)	0.253	0.615
Drinking [n(%)]	11 (7.59%)	11 (8.53%)	0.082	0.775
Hypertension [n(%)]	22 (15.17%)	20 (15.50%)	0.006	0.939
Diabetes [n(%)]	12 (8.28%)	10 (7.75%)	0.025	0.873
ASA classification [n(%)]			0.509	0.476
I	86 (59.31%)	71 (55.04%)		
II	59 (40.69%)	58 (44.96%)		
Disease type [n(%)]			1.412	0.494
Uterine fibroids	59 (40.69%)	61 (47.29%)		
Adenomyosis	36 (24.83%)	26 (20.16%)		
Endometriosis	50 (34.48%)	42 (32.56%)		

BMI, body mass index; ASA, American society of anesthesiologists.

### 3.2 Postoperative recovery

Comparison of postoperative recovery indicators between the conventional group and the prehabilitation group demonstrated significant differences across all measured parameters. Compared with the conventional group, patients in the prehabilitation group showed significantly shorter times to first postoperative anal exhaust (22.63 ± 4.38 h vs. 24.47 ± 5.44 h, t = 3.091, P = 0.002), first bowel movement (61.08 ± 6.23 h vs. 63.26 ± 7.21 h, t = 2.663, P = 0.008), and first ambulation (18.45 ± 3.74 h vs. 22.34 ± 4.09 h, t = 8.187, P < 0.001). Additionally, postoperative hospital stay duration was significantly shorter for patients in the prehabilitation group (4.39 ± 0.71 days) than those in the conventional group (6.95 ± 1.03 days), with a notably significant t-value of 24.236 (P < 0.001, **Table 2**).

**Table 2:** Comparison of postoperative recovery indicators between two groups.

Parameter	Conventional group (n=145)	Prehabilitation group (n=129)	t	P
First postoperative anal exhaust time (h)	24.47 ± 5.44	22.63 ± 4.38	3.091	0.002
First bowel movement time (h)	63.26 ± 7.21	61.08 ± 6.23	2.663	0.008
First postoperative ambulation time (h)	22.34 ± 4.09	18.45 ± 3.74	8.187	< 0.001
Postoperative hospital stays (d)	6.95 ± 1.03	4.39 ± 0.71	24.236	< 0.001

Comparison of VAS scores between the conventional group and the prehabilitation group revealed no significant difference in preoperative VAS scores (P = 0.617). However, significant differences were observed at several postoperative time points. At 6 h postoperatively, the prehabilitation group reported significantly lower VAS scores (4.81 ± 0.99 points) than the conventional group (5.16 ± 0.85 points), with a t-value of 3.075 (P = 0.002). At 24 h postoperatively, the prehabilitation group also showed significantly lower VAS scores (3.95 ± 0.72 points) than the conventional group (4.18 ± 0.62 points) (t = 2.729, P = 0.007). This trend continued at 48 h postoperatively, where the prehabilitation group again revealed lower VAS scores (2.87 ± 0.54 points) compared to the conventional group (3.01 ± 0.46 points), with a t-value of 2.334 (P = 0.020, **Figure 1**).

**Figure 1:**
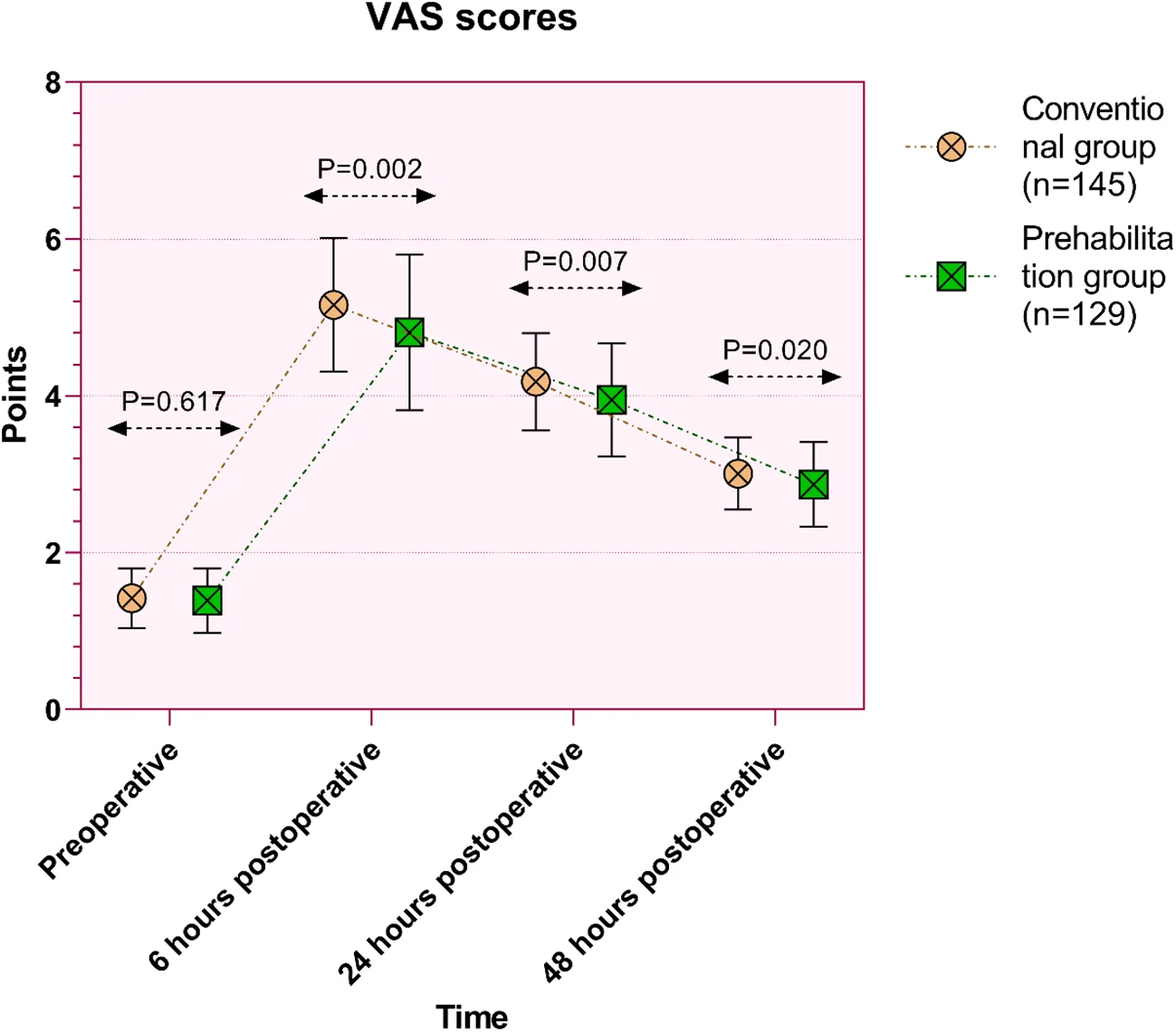
Comparison of VAS scores between two groups (points). VAS, visual analogue scale.

### 3.3 Venous thromboembolism

Regarding the comparison of Caprini risk levels and lower-extremity DVT incidence between the conventional and prehabilitation groups, no significant difference was observed in the distribution of Caprini risk levels preoperatively (P = 0.995). However, at 24 h postoperatively, the prehabilitation group demonstrated a significantly improved risk profile, with a higher proportion of patients in the low and moderate risk categories and a lower proportion in the high and very high-risk categories compared with the conventional group (χ² = 33.576, P < 0.001). Furthermore, the incidence of postoperative lower-extremity DVT was significantly lower in the prehabilitation group than in the conventional group (χ² = 12.625, P < 0.001, **Table 3**).

**Table 3:** Comparison of Caprini risk and lower extremity DVT between two groups [n(%)].

Parameter	Conventional group (n=145)	Prehabilitation group (n=129)	χ^2^	P
Caprini risk level (preoperative)			0.074	0.995
Low risk	12 (8.28%)	10 (7.75%)		
Moderate risk	28 (19.31%)	26 (20.16%)		
High risk	78 (53.79%)	70 (54.26%)		
Very high risk	27 (18.62%)	23 (17.83%)		
Caprini risk level (24 hours postoperative)			33.576	< 0.001
Low risk	10 (6.90%)	24 (18.60%)		
Moderate risk	24 (16.55%)	50 (38.76%)		
High risk	78 (53.79%)	42 (32.56%)		
Very high risk	33 (22.76%)	13 (10.08%)		
Lower extremity DVT incidence	21 (14.48%)	3 (2.33%)	12.625	< 0.001

DVT, deep vein thrombosis.

### 3.4 Serum indicators

In the comparison of coagulation function between the conventional and prehabilitation groups, several significant postoperative differences were observed. For APTT, no significant difference was noted preoperatively (P = 0.528); however, at 24 h postoperatively, APTT showed a significant increase in the prehabilitation group (t = 2.347, P = 0.020) compared with the conventional group. Similarly, PT showed no significant preoperative difference (P = 0.857) but was significantly higher postoperatively in the prehabilitation group (t = 2.442, P = 0.015). FIB levels did not show significant differences preoperatively (P = 0.571). Postoperatively, FIB levels were significantly lower in the prehabilitation group (t = 3.097, P = 0.002). Additionally, D-D levels showed no significant difference preoperatively (P = 0.471), but were significantly lower 24 h postoperatively in the prehabilitation group (t = 19.134, P < 0.001, **Table 4**).

**Table 4:** Comparison of coagulation function between two groups.

Parameter	Conventional group (n=145)	Prehabilitation group (n=129)	t	P
APTT (s)				
Preoperative	29.56 ± 2.81	29.78 ± 2.91	0.632	0.528
24 hours postoperative	28.21 ± 2.63	28.95 ± 2.57	2.347	0.020
PT (s)				
Preoperative	12.38 ± 1.12	12.41 ± 1.15	0.180	0.857
24 hours postoperative	11.74 ± 1.08	12.05 ± 1.03	2.442	0.015
FIB (g/L)				
Preoperative	3.12 ± 0.56	3.08 ± 0.54	0.567	0.571
24 hours postoperative	3.85 ± 0.66	3.62 ± 0.58	3.097	0.002
D-D (μg/L)				
Preoperative	245.68 ± 78.42	238.94 ± 75.63	0.722	0.471
24 hours postoperative	685.37 ± 124.56	426.82 ± 98.74	19.134	< 0.001

APTT, activated partial thromboplastin time; PT, prothrombin time; FIB, fibrinogen; D-D, D-dimer.

In the comparison of inflammatory response indicators between the conventional and prehabilitation groups, no significant differences were observed preoperatively for CRP (P = 0.279) and IL-6 (P = 0.394). However, significant differences were noted 24 h postoperatively.

CRP levels were significantly lower in the prehabilitation group than in the conventional group (t = 2.826, P = 0.005). Similarly, IL-6 levels were also significantly lower in the prehabilitation group compared with the conventional group at 24 h postoperatively (t = 2.788, P = 0.006, **Figure 2**).

**Figure 2:**
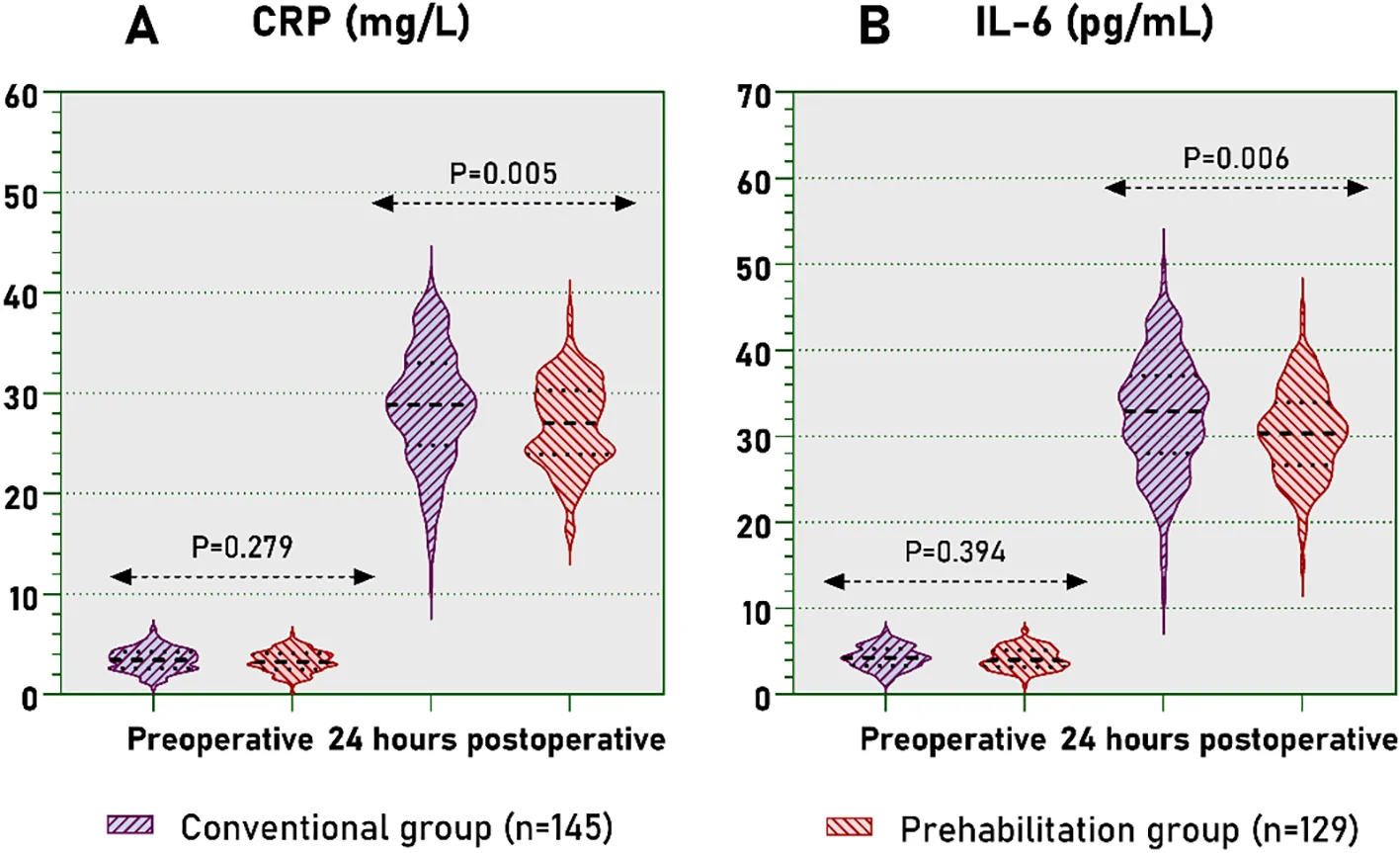
Comparison of inflammatory response between two groups. **(A)** CRP (mg/L); **(B)** IL-6 (pg/mL). CRP, C-reactive protein; IL-6, interleukin-6.

### 3.5 Complications

In the comparison of complication incidence between the conventional and prehabilitation groups, the overall incidence of complications was significantly lower in the prehabilitation group than in the conventional group (χ² = 5.484, P = 0.019). Specifically, the prehabilitation group showed decreased incidences of wound infection, urinary tract infection, subcutaneous emphysema, and shoulder-back pain (**Table 5**).

**Table 5:** Comparison of complications incidence between two groups [n(%)].

Parameter	Conventional group (n=145)	Prehabilitation group (n=129)	χ^2^	P
Complications incidence	28 (19.31%)	12 (9.3%)	5.484	0.019
Wound infection	6 (4.14%)	2 (1.55%)		
Urinary tract infection	8 (5.52%)	4 (3.1%)		
Subcutaneous emphysema	5 (3.45%)	2 (1.55%)		
Shoulder-back pain	9 (6.21%)	4 (3.1%)		

### 3.6 Multivariate logistic regression analysis

The multivariate logistic regression analysis identified significant risk factors for postoperative lower-extremity DVT occurrence in patients undergoing TLH. The multimodal prehabilitation program was identified as an independent protective factor (OR = 0.223, 95% CI: 0.099–0.502, P < 0.001). It is important to note that this protective effect reflects the composite impact of the entire prehabilitation bundle, as the program components were delivered as an integrated package and could not be analyzed separately due to perfect collinearity with group allocation. Conversely, a high-risk Caprini score (≥ high risk) (OR = 2.482, 95% CI: 1.791–4.271, P = 0.001) and elevated postoperative D-D levels (OR = 1.020, 95% CI: 1.007–1.034, P = 0.003) were significant independent risk factors for DVT occurrence (**Table 6**).

**Table 6:** Multivariate logistic regression analysis of risk factors for postoperative lower extremity DVT occurrence in patients undergoing TLH.

Parameter	Coefficient	Std error	Wald stat	P	OR	OR CI lower	OR CI upper
Multimodal prehabilitation program	-1.897	0.512	13.728	<0.001	0.223	0.099	0.502
Caprini risk level (≥high risk)	1.516	0.468	10.493	0.001	2.482	1.791	4.271
D-D	0.023	0.007	10.796	0.003	1.020	1.007	1.034

DVT, deep vein thrombosis; TLH, total laparoscopic hysterectomy; OR, odds ratio; CI, confidence interval; D-D, D-dimer.

## 4 Discussion

This study evaluated the impact of a multimodal prehabilitation program on postoperative recovery and VTE risk in patients undergoing TLH for benign conditions. The findings demonstrate that this preoperative intervention, which incorporates thrombosis risk assessment with tailored pharmacological prophylaxis, exercise training, nutritional support, and psychological preparation, was associated with improved recovery profiles, reduced VTE incidence, and modulated coagulation and inflammatory responses.

Faster gastrointestinal recovery, earlier ambulation, and shorter hospital stays in the prehabilitation group are consistent with enhanced recovery principles (20). After abdominal surgery, bowel function often recovers slowly due to anesthetics, surgical manipulation, and opioid use. Preoperative exercise training—especially ambulation-focused interventions—likely improved cardiovascular fitness and physical reserve. This enhancement enabled earlier postoperative mobilization and promoted gastrointestinal motility. Nutritional optimization, including anemia correction and protein supplementation, may have further supported tissue healing and functional recovery (21, 22). These findings are consistent with evidence from colorectal surgery studies, where prehabilitation programs incorporating exercise and nutrition have been found to reduce postoperative complications and hospital length of stay (23). Additionally, psychological interventions aimed at reducing anxiety may have contributed to improved outcomes, as lower preoperative anxiety has been correlated with reduced postoperative pain and improved recovery (24).

The prehabilitation group reported lower pain scores at multiple postoperative time points. Although the intervention did not directly target intraoperative analgesia, several mechanisms may explain this effect. Preoperative exercise and psychological preparation may modulate pain perception through central mechanisms, potentially raising pain thresholds (25). Better physical conditioning may also facilitate intraoperative positioning and reduce muscular strain (26). Nutritional support, particularly vitamin D supplementation, has been linked to improved musculoskeletal health and pain modulation. Overall, these findings align with research showing that preoperative interventions can influence postoperative pain and potentially reduce opioid requirements and their side effects (27).

The reduced incidence of postoperative lower-extremity DVT and the improved postoperative Caprini risk profile in the prehabilitation group represent central findings. The Caprini score, a validated VTE risk assessment tool, incorporates static patient factors and dynamic surgical factors (28). Preoperative administration of LMWH to high-risk patients represents a shift in the timing of prophylaxis, addressing the hypercoagulable state that begins intraoperatively and peaks in the early postoperative period (29). Despite the efficacy of standard postoperative prophylaxis, this intervention may not fully mitigate the thrombotic cascade initiated during surgery. Exercise training, specifically ankle pump exercises, likely contributed by enhancing lower-extremity venous return and reducing venous stasis, a key element of Virchow’s triad (30). This combined pharmacological and mechanical approach, initiated preoperatively, may provide more comprehensive blockage of thrombotic pathways. While existing literature supports the efficacy of LMWH and mechanical prophylaxis, evidence on optimal timing remains evolving. These findings indicate that, in high-risk gynecologic surgery patients, a prehabilitation strategy encompassing preoperative LMWH may provide additional VTE protection (15).

Serum marker analysis supports the biological plausibility of our clinical findings. Postoperatively, the prehabilitation group showed a coagulation profile suggesting a less pronounced hypercoagulable state, with higher APTT and PT values and lower FIB and D-D levels. Specifically, PT and APTT were significantly prolonged at 24 hours postoperatively, consistent with the residual anticoagulant effect of preoperative LMWH in high-risk patients. In contrast to the conventional group (which received LMWH postoperatively), the prehabilitation group achieved earlier systemic anticoagulation, which may have attenuated postoperative hypercoagulability. Elevated FIB and D-D levels, as markers of coagulation activation and fibrinolysis, typically increase after surgery and reflect thrombotic response to tissue trauma (31). The attenuated elevation of these markers indicates that preoperative interventions, particularly LMWH administration and exercise, may have tempered the surgical stress-induced coagulation cascade. Lower D-D levels have also been associated with reduced DVT risk. Furthermore, reduced postoperative CRP and IL-6 levels in the prehabilitation group indicate a blunted systemic inflammatory response (32). Surgical trauma triggers a strong inflammation reaction that contributes to pain, ileus, and pro-coagulant states. Exercise training has well-documented anti-inflammatory effects, while improved nutritional status can further modulate immune function (33). By mitigating this inflammatory surge, the prehabilitation program may have created a physiological environment less conducive to thrombotic events and other complications (34).

The lower overall complication rate in the prehabilitation group, including wound and urinary tract infections, subcutaneous emphysema, and shoulder-tip pain, highlights the broader benefits of preoperative optimization (14). These varied complications may all be influenced by preoperative physiological state. Improved nutritional status can improve immune competence and wound healing, potentially reducing infection risk (35). Additionally, better physical conditioning and respiratory training may help minimize referred shoulder pain associated with carbon dioxide insufflation (36). Multivariate analysis further confirmed the multimodal prehabilitation program as an independent protective factor against DVT, while high Caprini risk and elevated D-D were regarded as independent risk factors. These findings reinforce the clinical utility of these assessment tools in identifying patients who may benefit most from intensive prophylactic strategies.

Several limitations of this study should be acknowledged. First, the retrospective single-center design may introduce selection bias. Non-randomized allocation also raises the possibility of unmeasured confounding. Second, the difference in LMWH timing between groups represents a major confounding factor. This timing difference was perfectly collinear with group allocation, preventing adjustment as an independent variable in our multivariate model. Therefore, we cannot definitively determine whether the observed VTE reduction resulted from preoperative pharmacological prophylaxis alone, the composite effect of the entire prehabilitation bundle, or a synergistic combination. Third, the study population was limited to patients with benign disease and low anesthetic risk (ASA I–II), thus restricting the generalizability of the findings to higher-risk populations, such as those with malignancies or notable comorbidities. Fourth, the follow-up period for DVT detection was limited to 30 days post-discharge, potentially leading to the underdetection of late-occurring thrombotic events. Fifth, although the prehabilitation program was multimodal, the individual contribution of each component to the overall observed benefits is difficult to ascertain. Future prospective, randomized controlled trials involving larger and more diverse populations, as well as longer follow-up periods, are necessary to confirm these findings and evaluate the cost-effectiveness of implementing such programs.

## 5 Conclusion

This study demonstrates that a multimodal prehabilitation program, integrating preoperative thrombosis risk assessment with targeted pharmacological prophylaxis, exercise training, nutritional optimization, and psychological support, substantially improves postoperative recovery in patients undergoing TLH for benign diseases. The program was associated with accelerated recovery of gastrointestinal function, earlier ambulation, reduced postoperative pain scores, and shorter hospital stays. Importantly, the intervention effectively lowered the incidence of postoperative lower-extremity DVT, mitigated the surgery-induced hypercoagulable state as evidenced by improved coagulation parameters, and reduced the systemic inflammatory response. Overall, these findings support the implementation of multimodal prehabilitation as an effective strategy for enhancing recovery and reducing thrombotic risk in this patient population, supporting its integration into routine perioperative care pathways.

## Data Availability

The raw data supporting the conclusions of this article will be made available by the authors, without undue reservation.
